# Screening and Whole Genome Sequencing of SARS-CoV-2 Circulating During the First Three Waves of the COVID-19 Pandemic in Libreville and the Haut-Ogooué Province in Gabon

**DOI:** 10.3389/fmed.2022.877391

**Published:** 2022-05-17

**Authors:** Sonia Etenna Lekana-Douki, Nadine N'dilimabaka, Anthony Levasseur, Philippe Colson, Julia Cyrielle Andeko, Ornella Zong Minko, Octavie Banga Mve-Ella, Pierre-Edouard Fournier, Christian Devaux, Bertrand Mve Ondo, Falone Larissa Akombi, Laurianne Yacka Mouele Bolo, Audrey Michel Ngonga Dikongo, Abdoulaye Diané, Arsène Mabika Mabika, Jenny Francine Mathouet, Cresh Dzembo, Nick Chenis Atiga, Anicet Mouity Matoumba, Nal Kennedy Ndjangangoye, Ludivine Bréchard, Marielle Bedotto-Buffet, Joa Braithe Mangombi Pambou, Marisca Kandet Yattara, Elvire Anita Mbongo Nkama, Armel Mintsa Ndong, Ayola Akim Adegnika, Didier Raoult, Florence Fenollar, Jean-Bernard Lekana-Douki

**Affiliations:** ^1^Centre Interdisciplinaire de Recherches Médicales de Franceville (CIRMF), Franceville, Gabon; ^2^Département de Biologie, Faculté des Sciences, Université des Sciences et Techniques de Masuku, Franceville, Gabon; ^3^IHU-Méditerranée Infection, Marseille, France; ^4^Aix Marseille Univ, IRD, AP-HM, MEPHI, Marseille, France; ^5^Aix Marseille Univ, IRD, AP-HM, SSA, VITROME, Marseille, France; ^6^Centre National de la Recherche Scientifique (CNRS), Marseille, France; ^7^Unité Mixte de Recherche CIRMF-SSM, Libreville, Gabon; ^8^Hôpital d'instructions des Armées Akanda, Akanda, Gabon; ^9^Hôpital d'Instructions des Armées Omar Bongo Ondimba, Libreville, Gabon; ^10^Laboratoire National de Santé Publique, Libreville, Gabon; ^11^Centre de Recherches Médicales de Lambaréné (CERMEL), Lambaréné, Gabon; ^12^Institute of Tropical Medecine, University of Tübingen and German Center for Infectious Research (DZIF), Tübingen, Germany; ^13^Département de Parasitologie-Mycologie Médecine Tropicale, Faculté de Médecine, Université des Sciences de la Sante, Libreville, Gabon

**Keywords:** SARS-CoV-2, variant, whole genome sequencing, Libreville and Haut-Ogooué, Gabon

## Abstract

Since the onset of the COVID-19 pandemic, the SARS-CoV-2 viral dynamics in Africa have been less documented than on other continents. In Gabon, a Central African country, a total number of 37,511 cases of COVID-19 and 281 deaths have been reported as of December 8, 2021. After the first COVID-19 case was reported on March 12, 2020, in the capital Libreville, the country experienced two successive waves. The first one, occurred in March 2020 to August 2020, and the second one in January 2021 to May 2021. The third wave began in September 2021 and ended in November 2021. In order to reduce the data gap regarding the dynamics of SARS-CoV-2 in Central Africa, we performed a retrospective genotyping study using 1,006 samples collected from COVID-19 patients in Gabon from 2020 to 2021. Using SARS-CoV-2 variant screening by Real-Time Quantitative Reverse Transcription PCR (qRT-PCR) and whole genome sequencing (WGS), we genotyped 809 SARS-CoV-2 samples through qRT-PCR and identified to generated 291 new genomes. It allowed us to describe specific mutations and changes in the SARS-CoV-2 variants in Gabon. The qRT-PCR screening of 809 positive samples from March 2020 to September 2021 showed that 119 SARS-CoV-2 samples (14.7%) were classified as VOC Alpha (Pangolin lineage B.1.1.7), one (0.1%) was a VOC Beta (B.1.351), and 198 (24.5 %) were VOC Delta (B.1.617.2), while 491 samples (60.7%) remained negative for the variants sought. The B1.1 variant was predominant during the first wave while the VOC Alpha dominated the second wave. The B1.617.2 Delta variant is currently the dominant variant of the third wave. Similarly, the analysis of the 291 genome sequences indicated that the dominant variant during the first wave was lineage B.1.1, while the dominant variants of the second wave were lineages B.1.1.7 (50.6%) and B.1.1.318 (36.4%). The third wave started with the circulation of the Delta variant (B.1.617). Finally, we compared these results to the SARS-CoV-2 sequences reported in other African, European, American and Asian countries. Sequences of Gabonese SARS-CoV-2 strains presented the highest similarities with those of France, Belgium and neighboring countries of Central Africa, as well as West Africa.

## Introduction

Severe acute respiratory syndrome coronavirus 2 (SARS-CoV-2) was identified in Wuhan, China, at the end of 2019 (Wuhan-Hu-1 strain). This sarbecovirus rapidly spread worldwide causing a global pandemic recognized by the World Health Organization (WHO) on March 11, 2020 (1–3). In Africa, SARS-CoV-2 was first reported in February 2020, with the earliest diagnosed cases detected in Egypt and Nigeria. The first viruses circulating in Africa were considered to be of American and European origins. An African Task Force was rapidly set-up in partnership with the WHO, and most African countries were able to test for SARS-CoV-2 infection, although specific kits were in short supply. By April 2020, the African Centers for Disease Control and Prevention (Africa CDC) had reported 19,895 confirmed cases, including 1,017 deaths, from 52 African countries. However, compared to the global 7,700 genome sequences of SARS-CoV-2 available in GISAID at this date, the African continent has only reported 90 genome sequences from five countries (4, 5). In order to better understand the dynamics of the SARS-CoV-2 pandemic in Africa and worldwide, it is essential to increase international efforts to sequence the clades circulating in Africa and to assess the rate of population infected with these variants over time.

It is currently well established that SARS-CoV-2 has undergone several genetic modifications over time, leading to mutations or deletions located throughout its genome. Furthermore, these affect structural proteins such as the spike region encoded by the S gene. Some of these changes may have resulted in an increase in the global number of SARS-CoV-2 infections and the prevalence of COVID-19 cases. D614G, and N501Y substitutions in the spike protein have been correlated with increased virus replication and transmission, resulting in an increase in the number of COVID-19 cases (6, 7). Since 2020, several major viral variants have been reported in different countries (8–11). Despite the restrictive measures on international transport and the lockdown measures applied in most countries, the variants have continued to spread from one geographic region to another. Though they share a common ancestry (the Wuhan-Hu-1 strain), the SARS-CoV-2 clades that have spread worldwide in the past 2 years differ phylogenetically because of mutations within the genome, notably in the spike region. Thus, variants of concern (VOCs) and variants of interest (VOIs) were defined by the WHO, the CDC, and the COVID-19 Genomics UK (COG-UK) Consortium (12–14). VOI was defined as a variant with genetic modifications that affect the characteristics of the virus such as transmissibility, disease severity, immune escape and that cause significant community transmission resulting in an increase in the number of cases. VOC was defined as a VOI for which there is an increase in virulence, a significant reduction in neutralization by antibodies generated during previous infection or vaccination, reduced efficacy of treatments or vaccines, or diagnostic detection failures (15).

In September 2021, the four major VOCs were Alpha (Pangolin lineage B.1.1.7, V1, clade 20I), Beta (B.1.351, Nextstrain clade 20H), Gamma (P.1, V3, clade 20J) and Delta (B.1.617.2, clade 21A/ clade 21J), identified for the first time in the United Kingdom, South Africa, Brazil and India, respectively. Variants of interest emerged in the USA (Epsilon, B.1.427/9, clade 21C; Iota, B.1.526, clade 21F), Brazil (Zeta, P2), Peru (Lambda, C.37, clade 21G), Nigeria (Eta, B.1.525, clade 21D), India (Kappa, B.1.617.1, clade 21B) and the Philippines (Theta, P3, clade 21E) (16). The Alpha VOC contains three deletions del69-70HV, del144Y and seven mutations N501Y, A570D, D614G, P681H, T716I, S982, D1118H in the spike protein (17, 18).

The Alpha VOC was described to be more transmissible because of its ability to antagonize innate immunity (19). The amino-terminal domain (NTD) deletion was frequently associated with prolonged SARS-CoV-2 infection (20). Although it emerged in the second half of 2020 like the three other VOCs, the prevalence of the Alpha VOC gradually increased in the first quarter of 2021 and it became predominant in the USA and Europe from March 2021 (16). The Beta VOC has three Receptor-Binding Domain (RBD) mutations (K417N, E484K and N501Y), one amino-terminal domain (NTD) mutation and two deletions (L18F, del242), and has been associated with reduced vaccine efficacy (16). This variant rapidly spread in South-Africa, in South-Eastern Africa (Mozambique, Zambia, Botswana, Malawi, Zimbabwe) and in Europe between February 2021 and May 2021, though it was present on all continents (21). In the Republic of Djibouti, during the second wave of the pandemic, between February and May 2021, the South African variant (clade 20H) was linked with an increase in the number of severe forms of COVID-19 in patients (22). The Gamma variant (L18F, K417T, E484K, N501Y mutations) mostly spread in South America and Caribbean countries in June 2021 (16, 23). The mortality associated with this variant infection (VOC Gamma) appeared to increase 1.8 fold (23). The Delta VOC (B.1.617.2) (T95I, L452R, T478K mutations) spread on all continents including Africa from June 2021 and replaced the Alpha variant in Europe and the USA peaking in June, August and September 2021 (16, 24).

In order to contribute to international efforts characterizing the viral dynamics of SARS-CoV-2 and participate in the fight against the worldwide COVID-19 pandemic, the Gabonese government rapidly set up a genomic surveillance program. The main objective of this international collaboration was to reduce the data gaps and characterize SARS-CoV-2 strains circulating in Gabon, a Central African country. Monitoring the variants circulating in the country is crucial, as Gabon maintains numerous economic and cultural exchanges at the international level, in particular with Europe. Using SARS-CoV-2 variant screening by quantitative polymerase chain reaction (qPCR), and whole genome next-generation sequencing (WGS), we investigated the mutations and the SARS-CoV-2 clades in Gabon from 2020 to 2021. Furthermore, we compared the SARS-CoV-2 genomes identified in Gabon to those of viruses circulating in the Central African region.

## Materials and Methods

### Clinical Samples

Gabon is a country located in Central Africa with a total population of 2,226,000 inhabitants. The *Centre Interdisciplinaire de Recherches Médicales de Franceville* (CIRMF) received nasopharyngeal and oropharyngeal samples from several hospitals and health centers: the *Hôpital d'instruction des Armées Omar Bongo Onbimba* (HIAOBO), the *Hôpital d'instruction des Armées d'Akanda* (HIAA) in Libreville, and several health centers in the Haut-Ogooué province, in the two main cities Franceville and Moanda. The CIRMF performed the SARS-CoV-2 diagnosis by real-time Reverse Transcriptase Polymerase Chain reaction (qRT-PCR). The TIB MOLBIOL kit, which targeted the E and RdRp genes, then the Da An Gene Co., Ltd of Sun Yat-sen University kit which targeted the N and RdRp genes, the Sansure Biotech Inc kit which targeted the ORF1ab (Open Reading Frame 1) and N gene, the TaqPath COVID-19 kit from Thermofisher targeted ORF1ab, N and S genes, were successively used for diagnosis. A retrospective study was conducted in two main regions of Gabon, the capital Libreville and the Haut-Ogooué province during the two first epidemic waves, the two inter-waves and the beginning of the third wave (25). A total of 1,006 positive samples were collected in Libreville and the Haut-Ogooué province (in Franceville and Moanda).

### Screening SARS-CoV-2 Variants by RT-PCR

The strategy consisted in a first screening of VOCs and closely-related mutants of the Wuhan isolate for a large number of clinical samples (809), including those with a qPCR threshold value (Ct) > 30. Several qPCR were performed with primers and probes designed for specific regions of the SARS-CoV-2 genome involving characteristic mutations of VOCs and other variants. Four systems discriminated the VOCs: the UK Alpha variant (B.1.1.7, clade 20I) was targeted in the ORF8 gene, the Delta variant (B.1.617.2) was targeted by mutation P681R in the spike glycoprotein, the South African Beta variant (B.1.351, clade 20H) was targeted in the nsp2 gene, the Brazil Gamma variant (P1) was targeted in the N gene (22), (Supplementary Table 1). Two systems discriminated the reference strain NC_045512.2 (19A): the first targeted the RNA-dependent RNA polymerase (RdRp) gene, and the second, a spike glycoprotein fragment including 614D (Supplementary Table 1). Two other variants described in Marseille, Marseille-4 (B.1.160; 20A.EU2) and Marseille-1 (B.1.416), were targeted in ORF1ab genes (9, 26, 27). We screened 254 and 247 samples from the first and second wave, respectively. In addition, we screened 41 and 42 samples from the first and second inter-waves, respectively. Finally, 225 samples from September 2021 were screened for the third wave.

### SARS-CoV-2 Genome Sequencing

Whole genome next-generation sequencing was performed at IHU Méditerranée Infection, in France in order to collect additional information about the SARS-CoV-2 genetic polymorphism, to identify all the mutations present and the phylogenetic links of the viruses circulating in Libreville and the Haut-Ogooué province, and to compare them with the SARS-CoV-2 strains circulating in other regions. Samples with a Ct lower than 30 were selected for sequencing. Sixty-four samples from the first wave including 30 negative samples by qPCR were selected. One hundred and fifty-four including 32 positive VOCs Alpha by qPCR and 48 samples including 32 positive VOCs Delta by qPCR, from the second wave and the beginning of the third wave, were sequenced, respectively. In addition, one genome from the inter-wave 1 between the wave 1–2 and the genomes of 24 SARS-CoV-2 samples from the inter-wave 2 between the wave 2–3 were sequenced. Next-generation sequencing was performed on the NovaSeq 6000 instrument according to the Illumina COVID Seq protocol (Illumina Inc., San Diego, California, USA) and included the following steps: first strand cDNA synthesis from extracted viral RNA; cDNA amplification with two COVID Seq primer pools (28); fragmentation-tagging of PCR amplicons; clean up; PCR amplification (seven cycles) of tagmented amplicons; pooling, clean up, quantification and normalization of libraries; and finally, library sequencing on a NovaSeq 6000 sequencing system SP flow cell (Illumina Inc.). The final genome consensus sequences were generated with the Minimap2 software (29) by mapping on the SARS-CoV-2 reference genome GenBank accession number NC_045512.2 (Wuhan-Hu-1 isolate). A frequency of the majoritary nucleotide ≥70% and a depth ≥10X were applied. Mutation detection was performed with the freebayes tool (https://github.com/freebayes/freebayes) (https://arxiv.org/abs/1207.3907v2) using a score of mapping quality of 20. The genome sequences were classified into lineages using Phylogenetic Assignment of Named Global Outbreak LINeages (Pangolin) and Nextclade (30). Sequences described in the present study have been deposited on the GISAID sequence database (https://www.gisaid.org/) with accession numbers EPI_ISL_8886132 - EPI_ISL_8886408 (Supplementary Table 2).

### Phylogenetic Analysis

Phylogeny reconstruction of genome sequences was performed using the maximum likelihood method. The alignments were computed with MAFFT with default parameters. The study sequences were compared with those of the reference Wuhan-Hu-1 isolate (GenBank Accession no. NC_045512) and other available genomes. Three phylogenetic trees corresponding to the three waves and one phylogenetic tree corresponding to the VOC Delta inter-wave from October 2020 to December 2020 were built using the Jukes-Cantor model and a total of 79 genomes from African countries, 37 genomes from Europe, 36 genomes from Asia and seven genomes from America selected among GISAID data (31). GISAID sequences whose dates corresponded to the periods of the three waves and the two inter-waves were selected and used in the construction of the dataset of each phylogeny. The analysis were conducted in MEGA7.

## Results

### Retrospective Analysis of Available Data on Epidemiology of SARS-CoV-2 in Gabon and Countries From the Central African Sub-region

**Figure 1:**
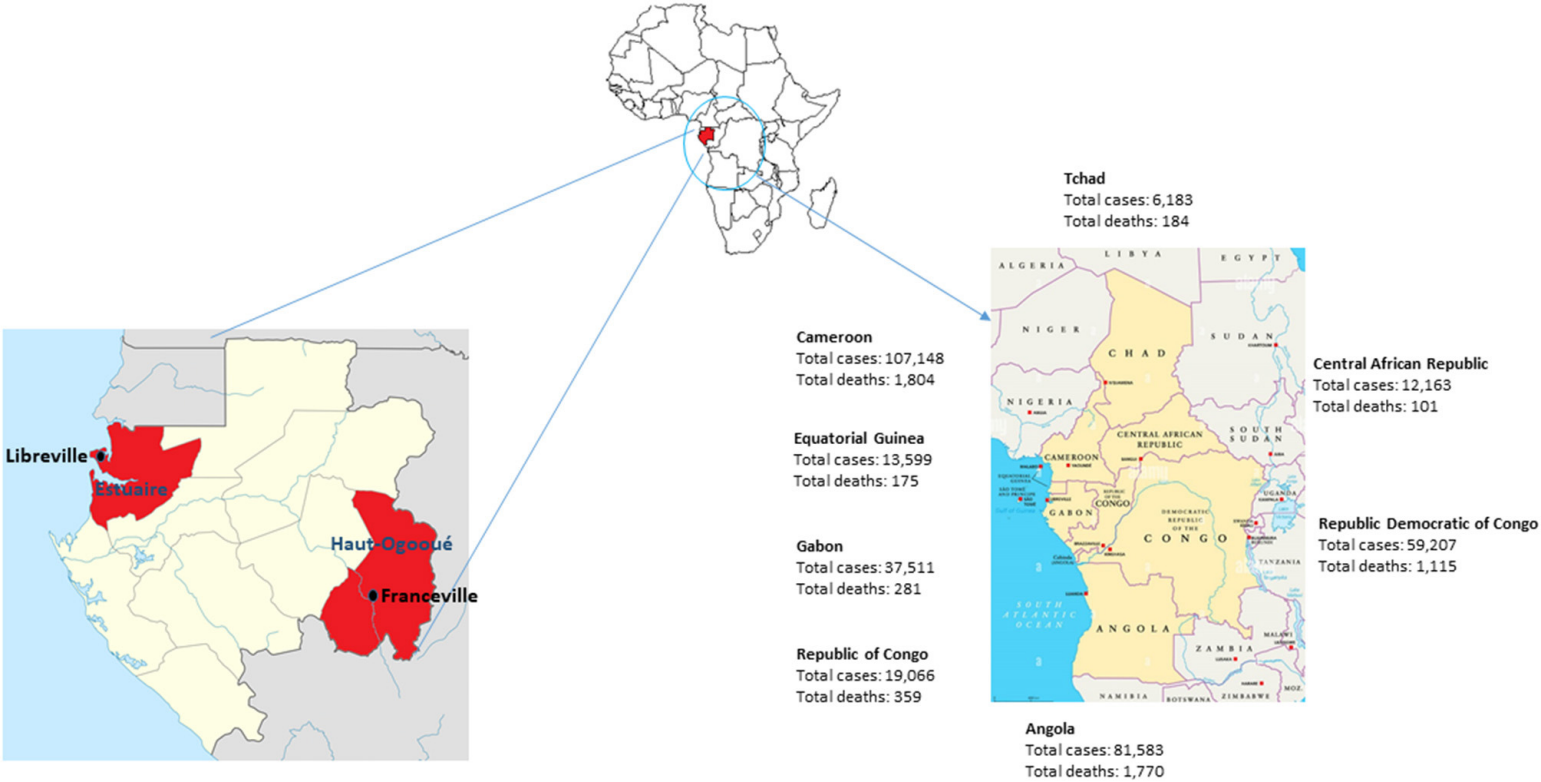
Map of Gabon and its neighboring countries in Central Africa. Number of COVID-19 cases and deaths in December 2021.

Gabon started diagnostic tests for COVID-19 on January 31, 2020. After the first case of COVID-19 was declared on March 12, 2020, in the capital Libreville, Gabon experienced two successive waves of the SARS-CoV-2 pandemic from March 2020 to August 2020, then from January 2021 to May 2021 (25). The third wave began in September 2021 and ended in November 2021. The total number of registered COVID-19 cases from the beginning of the pandemic until September 30, 2021 was 30,648 cases with a total of 191 deaths. As of December 8, 2021, there have been 37,511 cases of COVID-19 in Gabon and 281 deaths (Figure 1) (25).

A study carried out on samples from international travelers who arrived in Libreville in the first quarter of 2021 showed that 81% of SARS-CoV-2-positive individuals were infected with a variant of SARS-CoV-2. Among these, 41% had the Alpha variant, 32% had the Eta variant described in Nigeria, 6% had the Beta (1.351) variant from South Africa and 3% had the VOC Delta (32). In the sub-region, border countries, such as Cameroon, Republic of Congo and Equatorial Guinea, declared 107,148, 19,066 and 13,599 cases with 1,804, 359 and 175 dead respectively from the start of the pandemic until December 8, 2021 (Figure 1). These three countries declared the first case at the same period as Gabon in March 2020. However, Cameroon which is a country of 26,550,000 inhabitants experienced two waves at the same period as Gabon, while the Republic of Congo and Equatorial Guinea had a different progression of the virus (25). In the Republic of Congo (5,518,000 inhabitants), after the first wave took place between March 2020 and September 2020, the number of cases remained almost constant with a few weekly peaks in December 2020, January, April and May 2021. In Equatorial Guinea (1,403,000 inhabitants), the number of SARS-CoV-2 cases increased progressively until it reached a peak of 1,750 cases in August 2020. The second wave only started in September 2021 (25).

### Distribution of COVID-19 Cases

As indicated above, Gabon experienced the first wave of the SARS-CoV-2 pandemic from March 2020 to August 2020, a second wave from January 2021 to May 2021, and its third wave began in September 2021.

**Figure 2:**
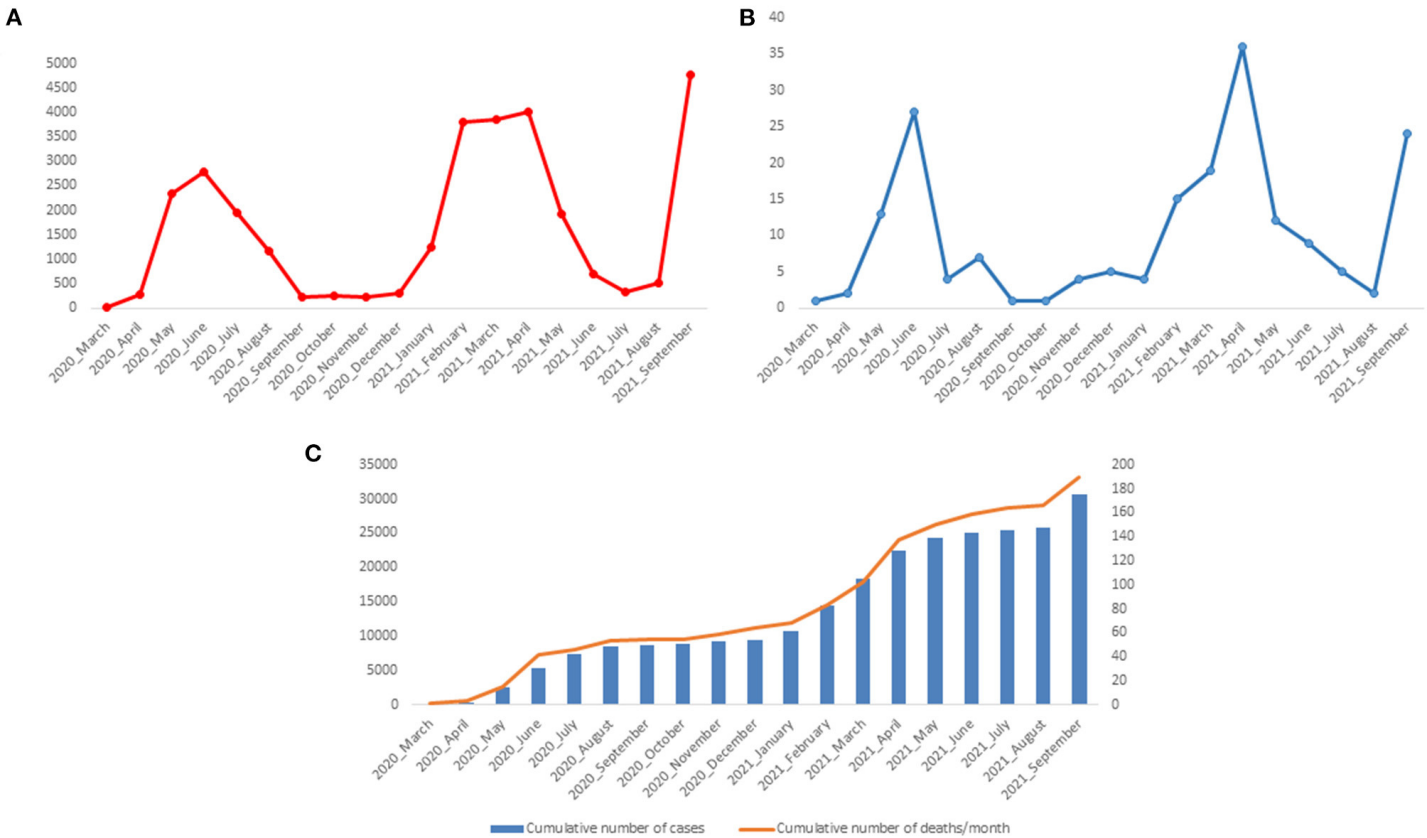
Number of positive cases of COVID-19 and deaths between March 2020 and September 2021 in Gabon. **(A)** Number of COVID-19 Cases per month; **(B)** Number of deaths associated with COVID-19 per month; **(C)** Cumulative number of COVID-19 cases and SARS-CoV-2 associated deaths.

During the first wave of the epidemic in Gabon, we diagnosed 8,505 cases of COVID-19. The number of cases reached a peak in June 2020 with 2,781 cases (Figure 2A). During the second wave, from January to May 2021, 14,868 people were diagnosed positive with SARS-CoV-2. The number of cases was 3,816, 3,862 and 4,007, in February, March and April 2021, respectively, the months corresponding to the peak of the second wave (Figure 2A). The number of deaths during the first two waves was 54 and 86, respectively. This number peaked in June 2020 (27 deaths) and April 2021 (36 deaths) (Figure 2B). The number of cumulative deaths increased proportionally to the number of cumulative cases (Figure 2C). In September 2021, at the beginning of the third wave, 4,760 new cases of COVID-19 and 24 deaths were recorded in Gabon.

### Identification of the VOCs Circulating in Libreville and the Haut-Ogooué Province by qPCR

A total of 809 positive samples were screened by qPCR from March 2020 to September 2021. In total, 119 (14.7%) belonged to the Alpha VOC (B.1.1.7), 1 (0.1%) to the Beta VOC (B.1.351), 198 (24.5%) to the Delta VOC and 491 (60.7%) were negative for the tested variants.

**Figure 3:**
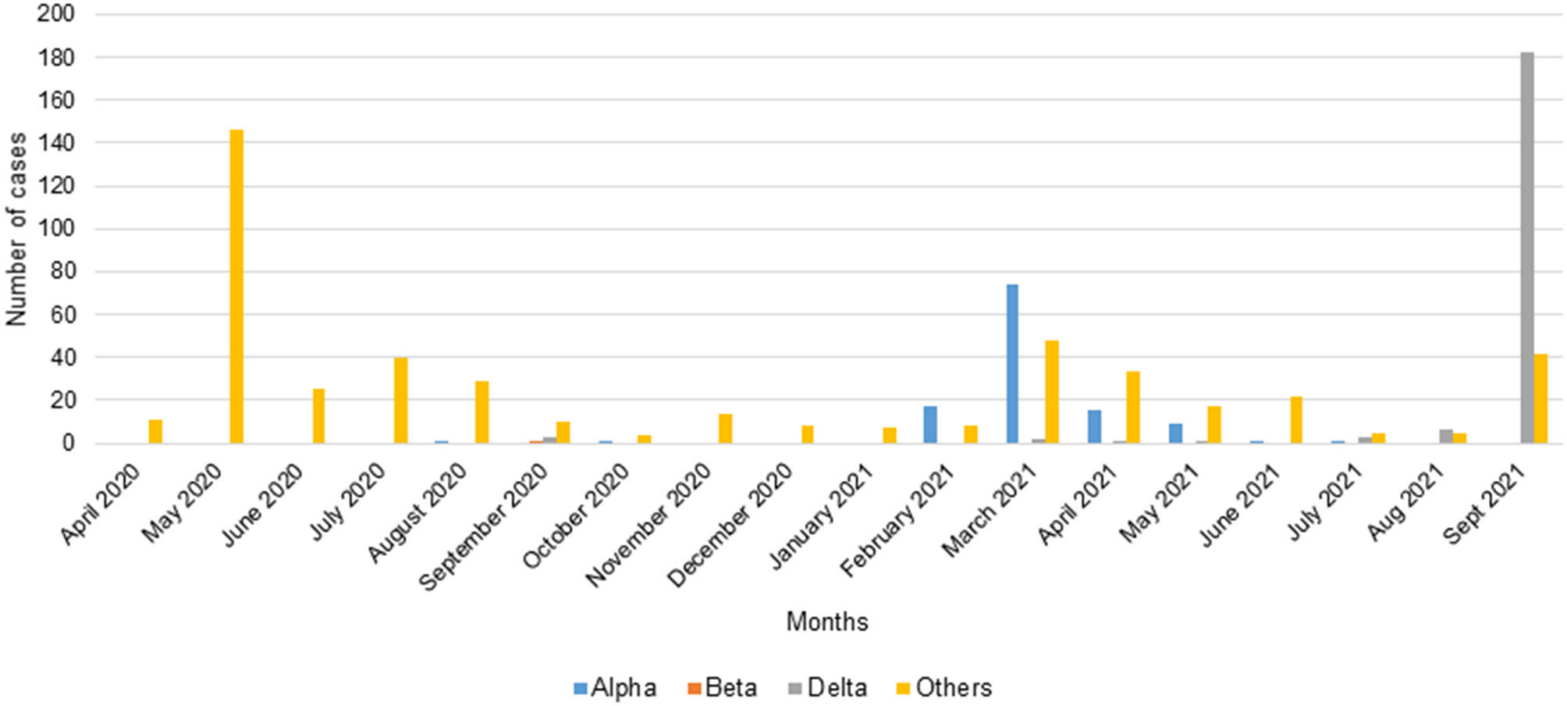
Number of cases of VOCs Alpha, Beta, Delta screened by qPCR.

The PCR carried out on the samples of the first wave using the primers (Wuhan1, Wuhan2) amplifying the reference strain showed that there was no amplification (Figure 3). At the end of the first wave, in August 2020, one sample (1/254) was identified as an Alpha VOC in Franceville. During the first inter-wave, 3/41 samples collected in September 2020 were positive for the Delta VOC while one collected in October 2020 was positive for the Alpha VOC and one collected in September 2020 was positive for the Beta VOC (Figure 3).

**Figure 4:**
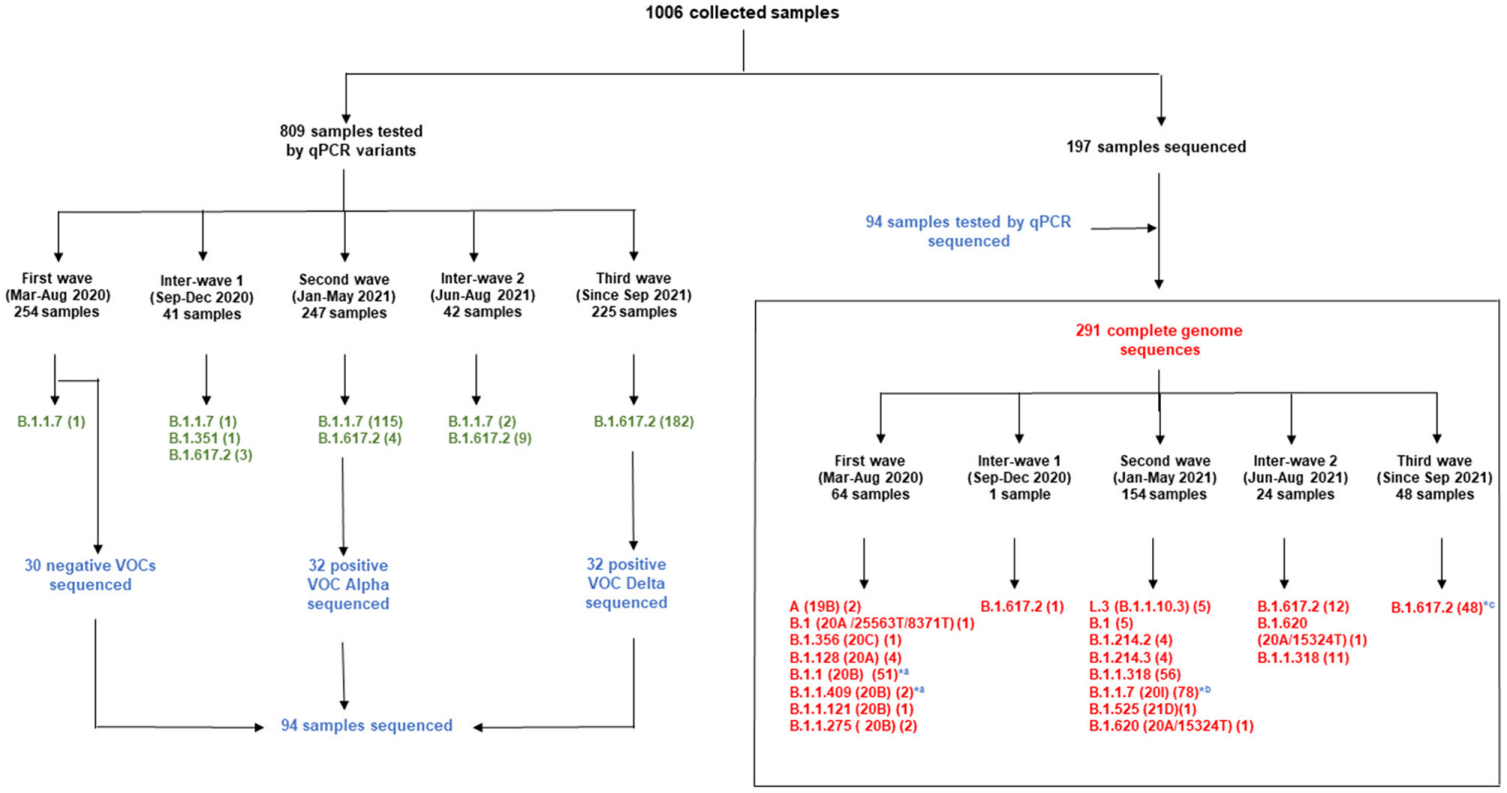
Distribution of samples screened by qPCR during the different waves and whole genome sequencing. qPCR results are in green; Samples first tested by qPCR and sequenced are in blue; Complete genome sequences are in red. *, genomes from samples tested by qPCR and sequenced; ^a^, first wave; ^b^, second wave; ^c^, third wave.

In the second wave, half of the samples belonged to the Alpha VOC (115/247). Although Alpha VOC was found in the first two waves, 96.6% (115/119) appeared in the second wave (Figure 4). In the second inter-wave, we found that 9/42 samples were positive for the Delta VOC (B.1.617.2) while 2/42 were positive for the Alpha VOC (Figure 3).

The third wave seemed to begin with a predominance of the Delta VOC (80.9%) (182/225) (Figure 4).

### Distribution of SARS-CoV-2 Variants During the Three Gabonese Waves

The lineage of each genome generated were mentioned in the supplementary material. The analysis of the sequences obtained showed that the first wave began in March 2020 with the circulation of viruses belonging to lineage A (Nextstrain clade 19B, GISAID clade S) (2/64), B.1 (Nextstrain clade 20A/25563T/8371T, GISAID clade G) (1/64) and B.1.356 (Nextstrain clade 20C, GISAID clade GH) (1/64). In this first wave, despite the four sequences of lineage B.1.128 (20A), we observed that the dominant sequences belonged to clade 20B (56/64; 87.5%), among which 51 (91.1%) belonged to lineage B.1.1 and the remaining to lineages B.1.1.409 (3.1%), B.1.1.121 (1.5%) and B.1.1.275 (3.1%) (Figure 4). Among the 30 negative VOCs from the first wave which were sequenced, 28 belonged to lineage B.1.1 and two belonged to lineage B.1.1.409 (Figure 4). All the genomes sequenced during the first wave carried the D614G spike mutation resulting from the non-synonymous A23403G, except for the two genomes of clade 19B (March and April 2020) and one virus of clade 20 B. The 19B viruses carried non-synonymous mutations T28144C and G28878A, leading to the substitutions L84S in the ORF8 and S202N nucleocapsid proteins, respectively (GISAID accession ID EPI_ISL_8886132, 56_Gabon and EPI_ISL_8886181, 211L_Gabon).

**Figure 5:**
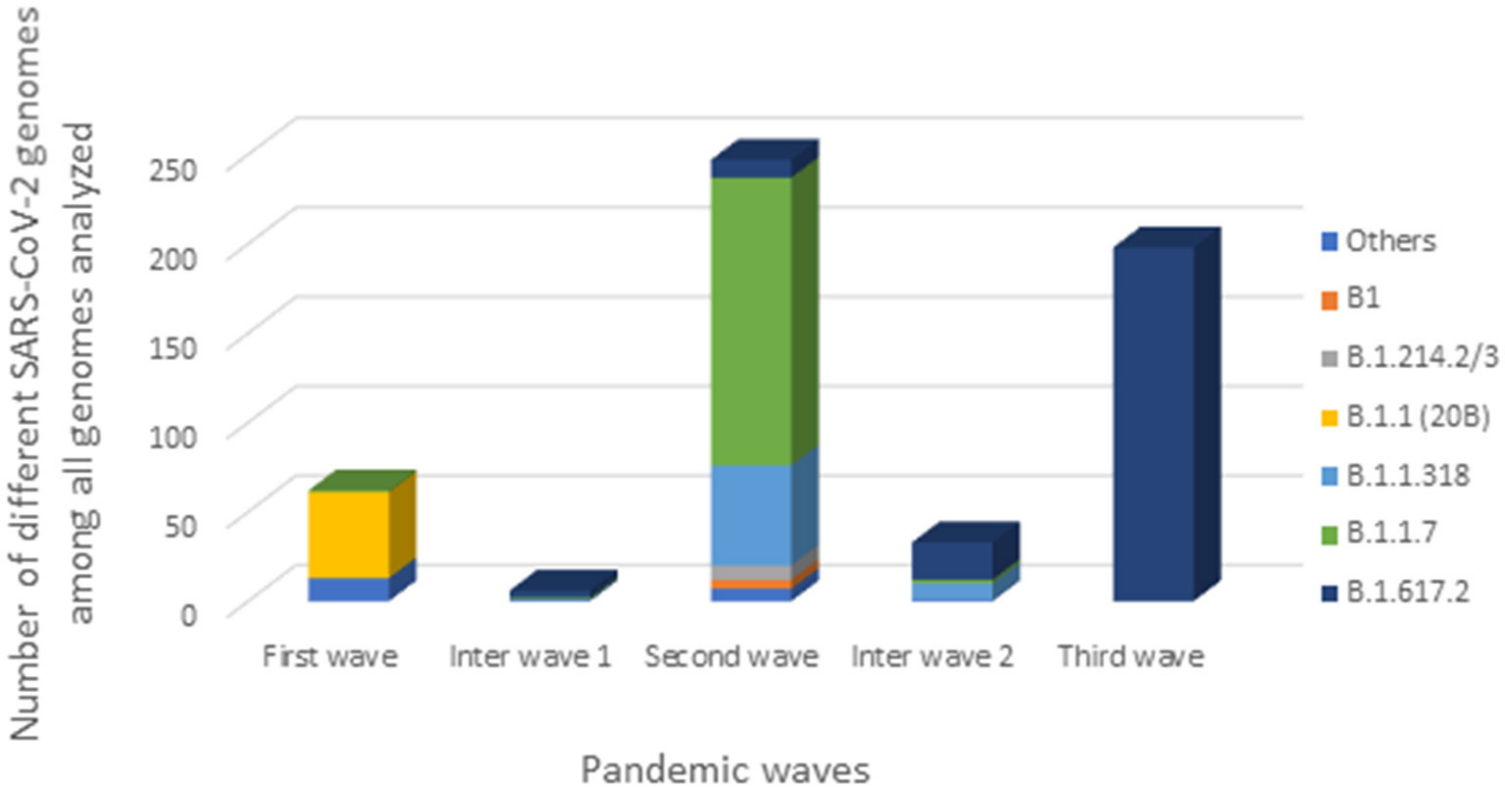
Distribution of SARS-CoV-2 strains during the three Gabonese pandemic waves. “Others” represented the others genomes we found: lineage A, B.1.356, B.1.128, B.1.1.409, B.1.1.121, B.1.1.275, L.3, B.1.620, B.1.525.

One genome obtained from a sample collected in December 2020, during the first inter-wave, belonged to VOC Delta. The genome of the 32 positive VOC alpha and the 32 positive VOC Delta by qPCR belonged to VOCs Alpha and Delta, respectively (Figure 4). Half of the genomes of the second wave (78/154) (50.6%) corresponded to the Alpha VOC (B.1.1.7; Nextstrain clade 20I; GISAID clade GR), while one third of the genomes belonged to lineage B.1.1.318 (56/154) (36.4%) (Figure 5). The other strains circulating at the same period belonged to lineages B.1.1.10.3 (clade L.3) (3.2%), B.1 (3.2%), B.1.214.2 (2.6%), and B.1.214.3 (2.6%), to lineage B.1.620 (0.6%) and to the Eta VBM (Variants Being Monitored) (B.1.525; 21D; clade G) (0.6%). In the second inter-wave, although we found lineages B.1.620 (4.2%) and B.1.1.318 (45.8%) were already present during the second wave, the predominant genomes were of VOC Delta (50.0%), which was exclusively found at the start of the third wave (Figure 5).

### Phylogenetic Analysis

**Figure 6:**
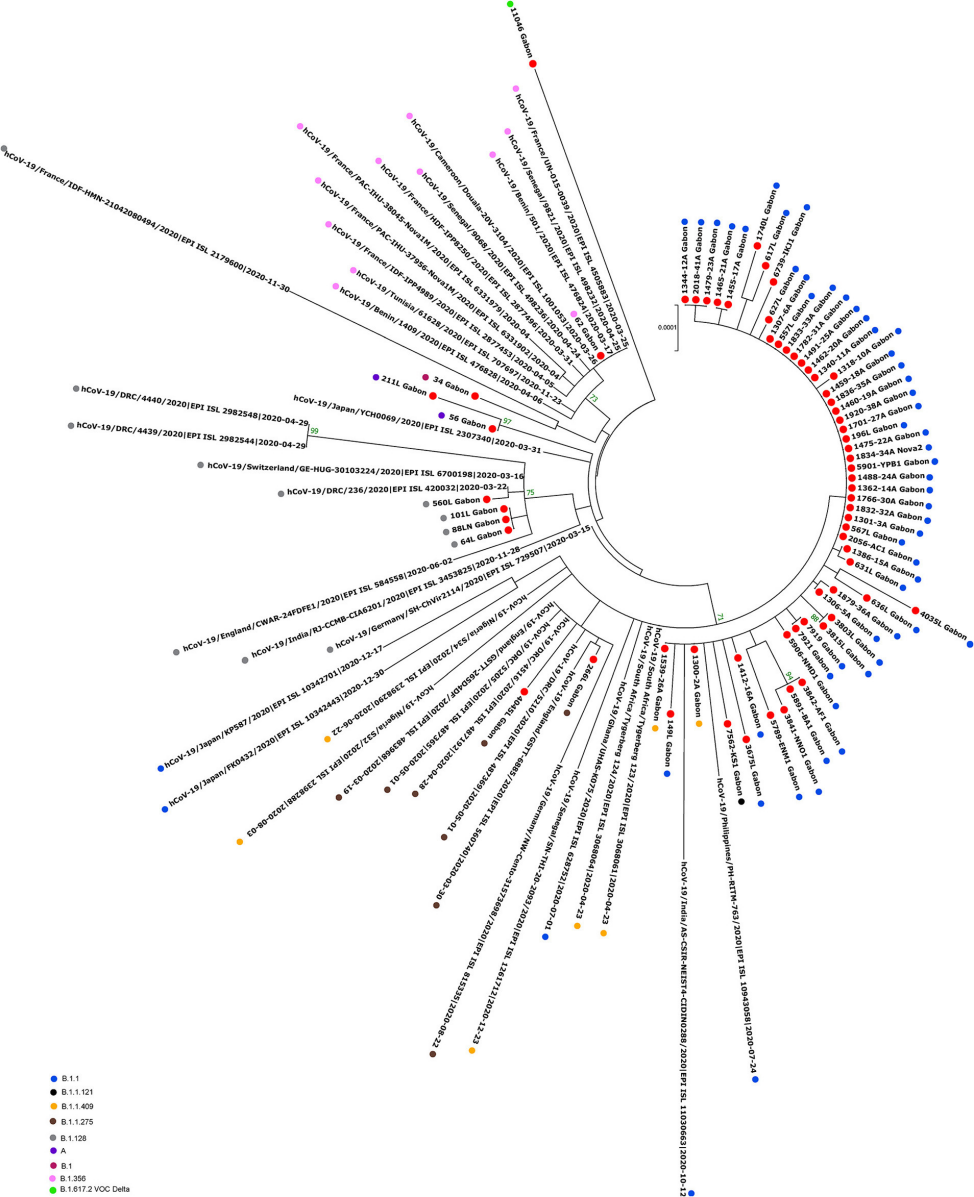
Molecular Phylogenetic analysis by Maximum Likelihood method using 65 sequences from strains circulating during the first wave and the first inter-wave in Gabon and 36 SARS-CoV-2 sequences available in GISAID. The evolutionary history was inferred by using the Maximum Likelihood method based on the Jukes-Cantor model. The tree with the highest log likelihood (−41732.01) is shown. Initial tree(s) for the heuristic search were obtained automatically by applying Neighbor-Join and BioNJ algorithms to a matrix of pairwise distances estimated using the Maximum Composite Likelihood (MCL) approach, and then selecting the topology with superior log likelihood value. The tree is drawn to scale, with branch lengths measured in the number of substitutions per site. The analysis involved 101 nucleotide sequences. All positions with <80% site coverage were eliminated. That is, fewer than 20% alignment gaps, missing data, and ambiguous bases were allowed at any position. There were a total of 28295 positions in the final dataset. Evolutionary analyses were conducted in MEGA7. The red circles correspond to the genomes identified in this study.

**Figure 7:**
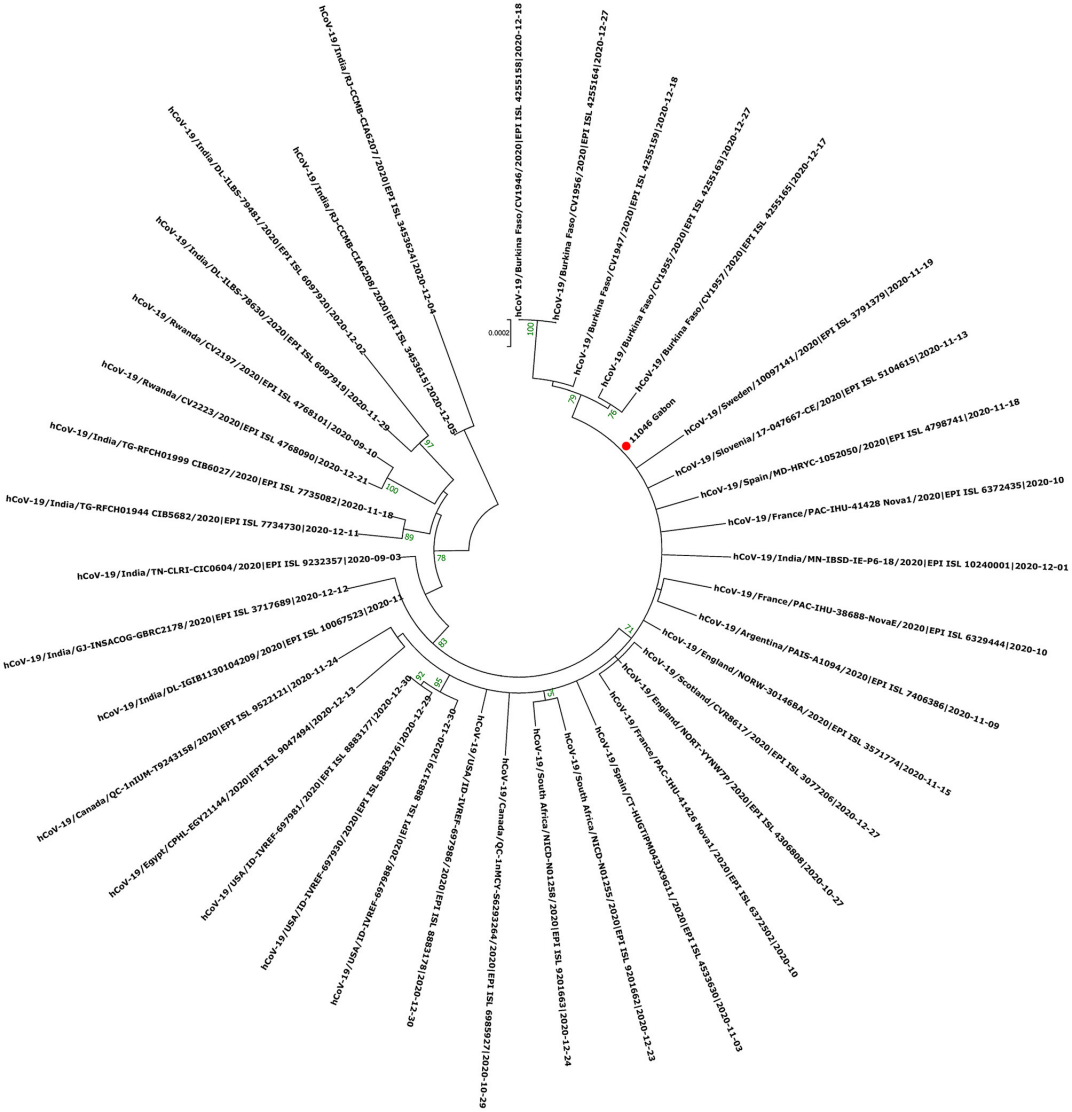
Molecular Phylogenetic analysis by Maximum Likelihood method using one sequence from VOC Delta circulating during the first inter-wave in Gabon and 37 SARS-CoV-2 sequences available in GISAID. The evolutionary history was inferred by using the Maximum Likelihood method based on the Jukes-Cantor model. The tree with the highest log likelihood (−41765.89) is shown. Initial tree(s) for the heuristic search were obtained automatically by applying Neighbor-Join and BioNJ algorithms to a matrix of pairwise distances estimated using the Maximum Composite Likelihood (MCL) approach, and then selecting the topology with superior log likelihood value. The tree is drawn to scale, with branch lengths measured in the number of substitutions per site. The analysis involved 38 nucleotide sequences. All positions with <80% site coverage were eliminated. That is, fewer than 20% alignment gaps, missing data, and ambiguous bases were allowed at any position. There were a total of 27791 positions in the final dataset. Evolutionary analyses were conducted in MEGA7. The red circle correspond to the genome identified in this study.

**Figure 8:**
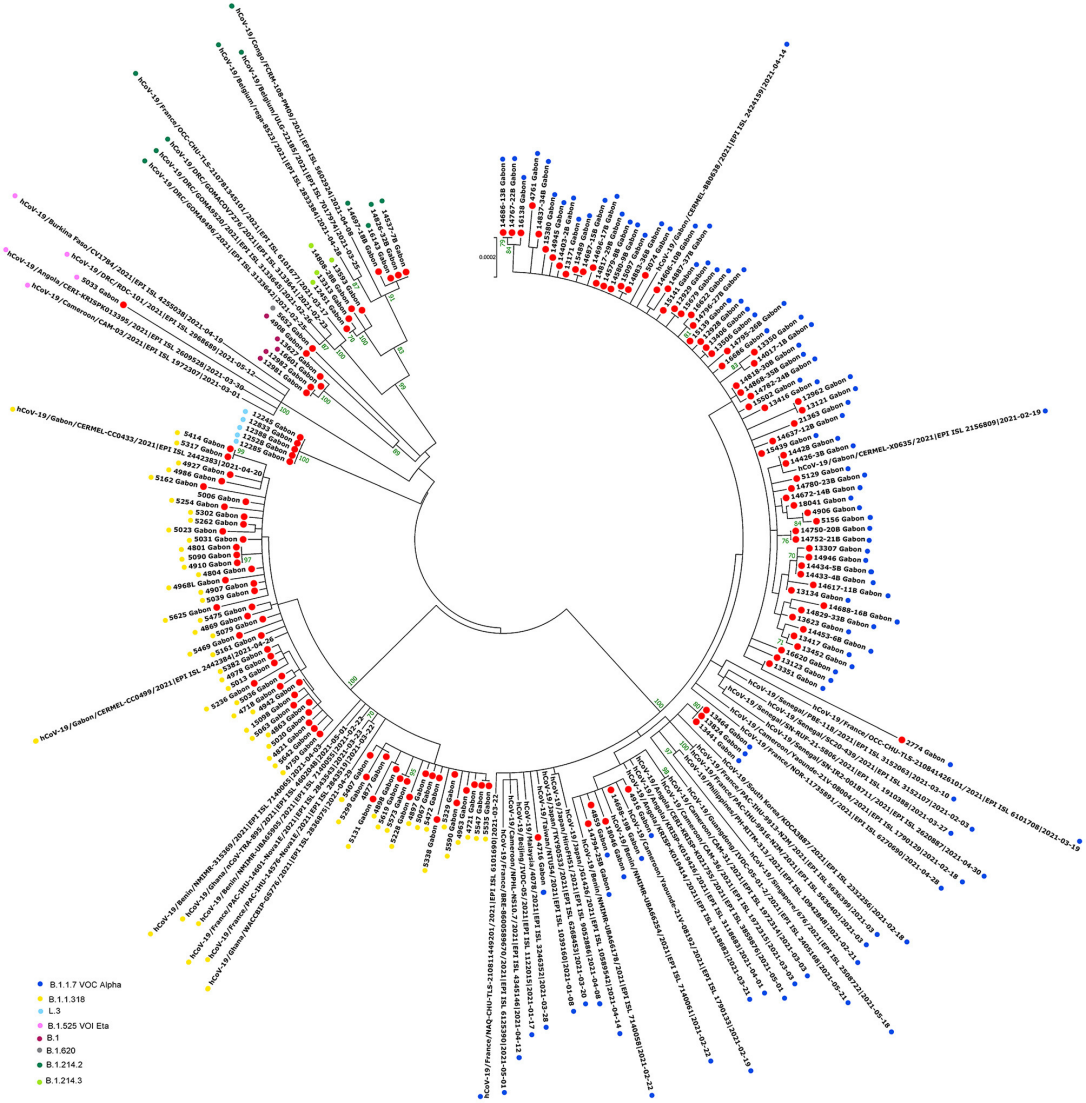
Molecular Phylogenetic analysis by Maximum Likelihood method using 154 sequences from strains circulating during the second wave in Gabon and 51 SARS-CoV-2 sequences available in GISAID. The evolutionary history was inferred by using the Maximum Likelihood method based on the Jukes-Cantor model. The tree with the highest log likelihood (−47967.94) is shown. Initial tree(s) for the heuristic search were obtained automatically by applying Neighbor-Join and BioNJ algorithms to a matrix of pairwise distances estimated using the Maximum Composite Likelihood (MCL) approach, and then selecting the topology with superior log likelihood value. The tree is drawn to scale, with branch lengths measured in the number of substitutions per site. The analysis involved 205 nucleotide sequences. All positions with <80% site coverage were eliminated. That is, fewer than 20% alignment gaps, missing data, and ambiguous bases were allowed at any position. There were a total of 28844 positions in the final dataset. Evolutionary analyses were conducted in MEGA7. The red circles correspond to the genomes identified in this study.

**Figure 9:**
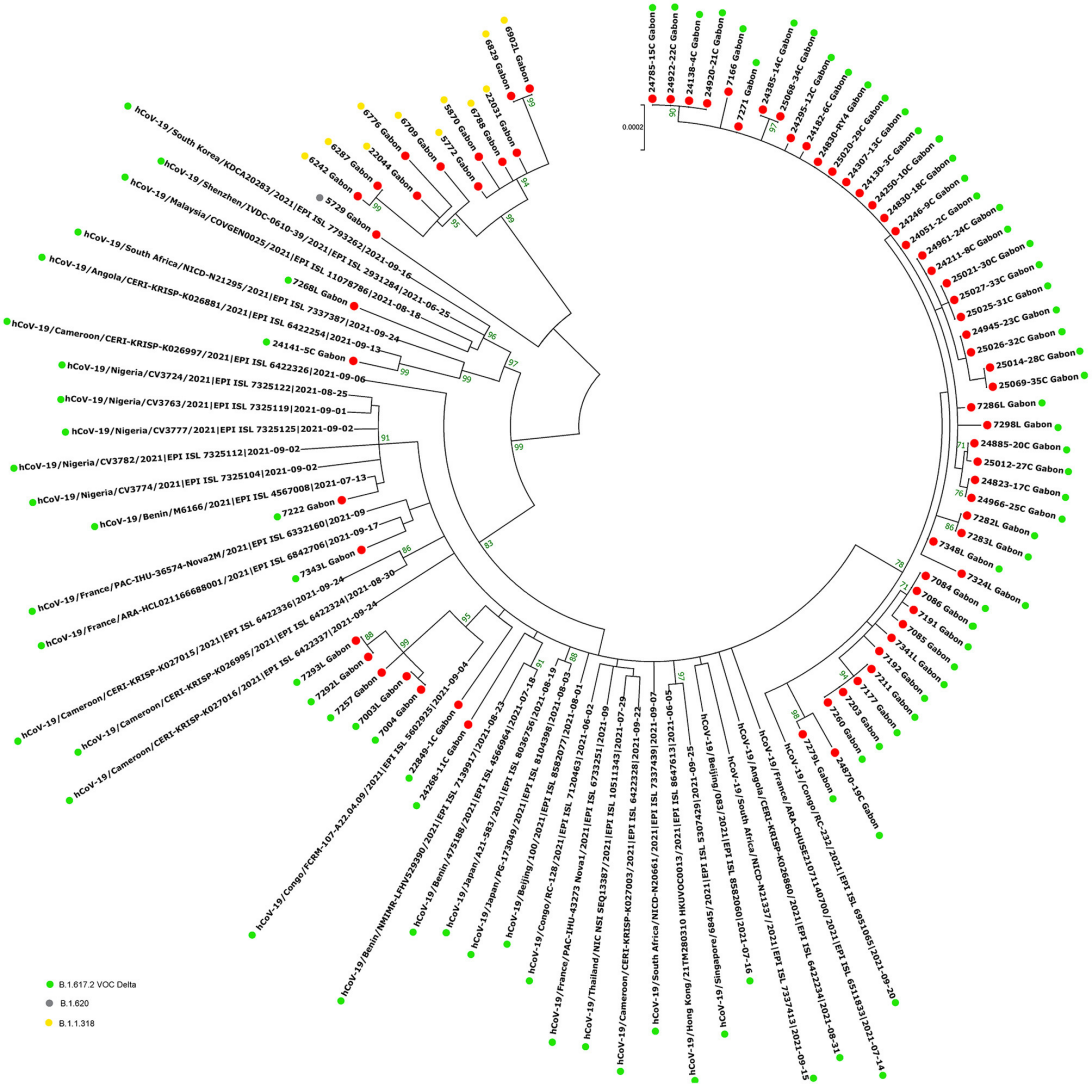
Molecular Phylogenetic analysis by Maximum Likelihood method using 72 sequences from strains circulating during the second inter-wave and the third wave in Gabon and 35 SARS-CoV-2 sequences available in GISAID. The evolutionary history was inferred by using the Maximum Likelihood method based on the Jukes-Cantor model. The tree with the highest log likelihood (−45247.72) is shown. Initial tree(s) for the heuristic search were obtained automatically by applying Neighbor-Join and BioNJ algorithms to a matrix of pairwise distances estimated using the Maximum Composite Likelihood (MCL) approach, and then selecting the topology with superior log likelihood value. The tree is drawn to scale, with branch lengths measured in the number of substitutions per site. The analysis involved 107 nucleotide sequences. All positions with <80% site coverage were eliminated. That is, fewer than 20% alignment gaps, missing data, and ambiguous bases were allowed at any position. There were a total of 28863 positions in the final dataset. Evolutionary analyses were conducted in MEGA7. The red circles correspond to the genomes identified in this study.

The phylogenetic data made it possible to compare the genomes of Gabon with those of neighboring countries and other African, European, American and Asian countries. However, they did not allow to certify their origin, which required a phylodynamic analysis. Phylogenetic analyses were conducted by building four phylogenetic trees, one for each of the three pandemic waves and the inter-wave 1 in Gabon. The first tree was built from 10 sequences of the first wave, from samples collected from March to April 2020. These sequences were distributed into four clusters, except for one named 4045L_Gabon for the month of July 2020. In contrast, the 54 other sequences from samples collected from May 2020 to July 2020 were grouped into a single cluster (Figure 6). Two sequences (211L_Gabon and 56_Gabon) were phylogenetically close to those of Wuhan, four others (560L_Gabon, 101L_Gabon, 88L_Gabon, 64L_Gabon) formed a cluster belonging to clade 20A (B.1.128) and were close to sequences from the DRC (EPI_ISL_2982544, EPI_ISL_2982548). The sequences named 34_Gabon and 62_Gabon have a strong similarity with sequences from France (EPI_ISL_2179600, EPI_ISL_6331904, EPI_ISL_6331979). The last one forms a cluster with sequences of clade 20C (Figure 6). Only two sequences, one from samples collected in April 2020 (266L_Gagon) and the other in July 2020 (4045L_Gabon), which belong to lineage B.1.1.275, were close to sequences of the DRC (EPI_ISL_487192) and England (EPI_ISL_560740). One isolated sequence (11046_Gabon) from a sample collected in December 2020 did not cluster with any others sequences (Figure 6). It was assigned to VOC Delta according Nextclade (Figure 4). The phylogenetic tree composed of 37 Delta VOC genomes from samples from the African, European, Asian and American continents, in the inter-wave between October and December 2020, showed that this genome is phylogenetic close to the others VOC Delta genomes (Figure 7). The second tree, corresponding to the second wave, revealed that the 78 sequences of the dominant Alpha variant were distributed in four distinct clusters. A group of 69 sequences formed a cluster with sequences from France (NOR-11735891, EPI_ISL6270690), Senegal SN (EPI_ISL_1910388, EPI_ISL_2620887) and also two other sequences from another Gabonese study in the region of Lambaréné (EPI_ISL_2156809, EPI_ISL_2424159) (Figure 8). The second cluster was made of sequences from Benin NMIMR (EPI_ISL_7140058, EPI_ISL_7140061), the third cluster was composed of sequences from Marseille (EPI_ISL_5636399 and EPI_ISL_5636402 belonging to the Alpha VOC). Finally, one sequence (ID4746) formed a cluster with sequences from France (EPI_ISL_6101690 belonging to the Alpha variant) and Cameroon NPHL (EPI-ISL_4345146). Two groups of four sequences belonged to the Belgian lineage B.1.214 (20A): the first had sequence homologies with two sequences from Belgium (EPI_ISL_2833384, EPI_ISL_7017874) and the other with sequences of the DRC (EPI_ISL_3133642, EPI_ISL_3133675) (Figure 8). The combined 56 sequences of lineage B.1.1.318 formed a cluster with two sequences of Lambaréné (EPI_ISL_2442383, EPI_ISL_2442384) and presented sequence homologies with two Ghanaian sequences (EPI_ISL_2836875, EPI_ISL_4602048) (Figure 8). We found one sequence belonging to the Eta VBM (Variants Being Monitored) (B.1.525), it presented homologies with a sequence of Cameroon CAM-03 (EPI_ISL_1972307). The third tree corresponding to the second interwave and the beginning of the third-wave revealed that 64 sequences belonged to lineage B.1.1.318 (9 sequences) and the Delta VOC (B.1.617.2) (55 sequences) which were distributed into seven clusters (Figure 9). Eighty percent (80%) (45/55) of the B.1.617.2 genome from Gabon formed a cluster with a sequence from the Republic of the Congo (EPI_ISL_6951065), five were clustered with another Congolese sequence (EPI_ISL_5602925) and one matched with sequence EPI_ISL_6951070 from Republic of the Congo. In addition, two sequences were clustered with sequences from Benin (EPI_ISL_4567008, EPI_ISL_7139917) and two others had sequence homologies with a French sequence (EPI_ISL_6842706) and an Angolan sequence (EPI_ISL_6422254) (Figure 9).

## Discussion

This study provided important new data on the lineages identified during the first three waves of COVID-19 in Gabon. The predominant variants were lineage B.1.1, VOC Alpha and VOC Delta during the first, second and third wave, respectively. A total of 1006 samples were collected for genotyping and sequencing. Eight hundred nine samples from the three waves were screening for VOCs Alpha, Beta, Gamma And Delta. Genome sequencing of the 30 negative VOCs from the first wave revealed that 28 were lineage B.1.1 and two were lineage B.1.1.409 (Figure 4). This explains the results of the qPCR showing the absence of amplification for the PCR systems amplifying VOCs, the Marseille-4 and Marseille-1 variants and Wuhan-1 and 2, except for one sample. The 32 positive VOC alpha and the 32 positive VOC Delta by qPCR which were sequenced belonged to VOCs Alpha and Delta, respectively (Figure 4). These results showed that genome sequencing corroborated the results of genotyping by qPCR.

The phylogenetic analysis of the 64 whole genomes revealed that 87.5% (56/64) of the SARS-CoV-2 strains belonged to clade 20B during the first wave. Only 1/254 sample was of the Alpha variant. This sample was collected in August 2020. A study reported that during this period VOCs were not predominant. However, it showed that the Alpha variant began to circulate as early as June 2020 (16). The 4 sequences of lineage B.1.128 (20A) were mainly reported in Canada (94% of the sequences) but also in Europe, particularly in Switzerland and France. In addition, it has been reported in the DRC, a neighboring country of Gabon (21). Phylogenetic analysis showed sequence homologies with sequences from DRC coming from samples collected in the same period (April 2020). This suggests a common entry point or circulation of the B.1.128 lineage during this month (Figure 6). In the Republic of Congo, the genomic surveillance of SARS-CoV-2 revealed that 9/11 sequences belonged to clade 20A while two sequences were part of clade 20C (33). Population movements and trade with the Republic of the Congo border could be another entry point of this strain. The tree in Figure 6 showed that at the beginning of the pandemic in Gabon, the circulating viruses belonged to clade 20A and 20C. However, in May, the dominant clade quickly became 20B (54 sequences). It suggests either a genetic evolution of the original viruses toward the 20B strains, or rather the entry of viruses belonging to lineage B.1.1 (clades 20B) toward the end of April 2020 or the beginning of May 2020. Lineage B.1.1.275, poorly reported, circulated in Angola and the DRC (21). The two Gabonese sequences (4045L_Gabon, 266L_Gabon) belonging to the B.1.1.275 lineage formed a cluster with the viruses from the DRC (Figure 6). It suggests a simultaneous circulation of these strains probably resulting from exchanges between Gabon and the DRC. In the first inter-wave, a VOC Delta genome was sequenced from a sample collected in December 2020 (11046_Gabon) and three samples were found positive by qPCR (Figure 4). According to the literature, like the VOC Alpha, the VOC Delta appeared as early as August 2020 (16). In order to investigate the genome 11046_Gab, a phylogenetic tree was built with 37 others VOC Delta genomes from the same period between October 2020 and December 2020) which confirmed the genome lineage (Figure 7). During the second wave, the VOC Alpha was predominant (50%), which corroborated the analyzes of a review showing the massive circulation of this lineage in the world during this period (16). The distribution of genomes of VOC Alpha into four clusters (Figure 8) showed that there were probably at least four entries points for this lineage in Gabon. The genomes of Lambaréré (EPI_ISL_2156809, EPI_ISL_2424159) (Figure 8) belonged to this main group, suggesting that it rapidly spread from one of these entry points around February 2021 to several regions of the country, especially Libreville, the Haut-Ogooué province and Lambaréné. A total of 56 sequences from samples collected between March and May 2021 belonged to lineage B.1.1.318 (Figure 4). Although present on all continents, lineage B.1.1.318 seems to have circulated mainly in the United States, Europe, and West Africa, notably in Nigeria, Benin, Ghana, Burkina-Faso but also in Cameroon, a neighboring country (21). It represented 16.2% of the virus strains found in a study carried out in Benin between January and April 2021 (34). These results corroborated another study carried out in the Lambaréné province in Gabon, which revealed the emergence of this lineage during this same period (35). The 56 sequences of lineage B.1.1.318 were divided into two groups, one group of 18 sequences and another including 38 sequences. These suggest several entry points for this lineage in Gabon, or genetic diversity. These Gabonese sequences presented homologies with those of Ghana (WACCBIP EPI_ISL_2836875, TRA-895 EPI_ISL_4602048) (Figure 8).

In conclusion, we observed a specificity of SARS-CoV-2 during the first three pandemic waves in Gabon. Indeed, the first wave was globally marked by the circulation of the B.1.1 (clade 20B) variant while the Alpha VOC, lineages B.1.1.7 and B.1.1.318, greatly circulated during the second wave. The third wave corresponded to the circulation of the Delta VOC (B.1.617). Although these phylogenetic data were not sufficient to clearly identify the origin of the genomes of Gabon, we showed homologies between them and those of France, the DRC, Congo, Senegal, Cameroon, Belgium, Benin and Ghana.

## Data Availability Statement

The original contributions presented in the study are publicly available. This data can be found here: GenBank; under accessions OM731935 - OM732217.

## Ethics Statement

Ethical review and approval was not required for the study on human participants in accordance with the local legislation and institutional requirements. Written informed consent from the participants or their legal guardian/next of kin was not required to participate in this study in accordance with the national legislation and the institutional requirements.

## Author Contributions

SL-D, NN'd, J-BL-D, AA, CD, DR, and FF contributed to the design of the study. MK, EM, and AMi took care of the COVID-19 patients and collected the samples. JM and OZ performed data collection. JA, OB, BM, FA, LY, AN, AD, AMa, JM, CDz, NA, AMo, NN, LB, and MB-B performed the laboratory analyses. LB, PC, AL, NN'd, and SL-D, generated and analyzed the virus sequences. SL-D and CD prepared the first draft of the manuscript. FF, DR, CD, P-EF, and SL-D supervised the work. P-EF, FF, DR, and J-BL-D obtained the funding for this study. All authors contributed to the article and approved the submitted version.

## Funding

This work was supported by the Gabonese government as part of the COVID-19 diagnostic and the genomic surveillance of variants. The study was also supported by the Institut Hospitalo-Universitaire (IHU) Méditerranée Infection, the French National Research Agency under the program ≪ Investissements d'avenir ≫, reference ANR-10-IAHU-03, the Région Provence Alpes Côte d'Azur and European funding FEDER PRIMMI (European Regional Development Fund-Plateformes de Recherche et d'Innovation Mutualisées Mediterranée). CD^1^ was supported by the French Centre National de la Recherche Scientifique.

## Conflict of Interest

The authors declare that the research was conducted in the absence of any commercial or financial relationships that could be construed as a potential conflict of interest.

## Publisher's Note

All claims expressed in this article are solely those of the authors and do not necessarily represent those of their affiliated organizations, or those of the publisher, the editors and the reviewers. Any product that may be evaluated in this article, or claim that may be made by its manufacturer, is not guaranteed or endorsed by the publisher.
