# Supplementary material for: Screening and Whole Genome Sequencing of SARS-CoV-2 Circulating During the First Three Waves of the COVID-19 Pandemic in Libreville and the Haut-Ogooué Province in Gabon
Source: Front Med (Lausanne). 2022 May 17;9:877391. doi: 10.3389/fmed.2022.877391 (PMC9152426; doi:10.3389/fmed.2022.877391)
Supplement: Supplementary file 1 [file Table_1.DOCX]

Supplementary Table 1 : qRT-PCR primers and probe

| SARS-CoV-2 strain | Target gene | Primers | Probe | Fragment  size (bp) | |
| --- | --- | --- | --- | --- | --- |
| Alpha variant | ORF8 | Sense : 5’-CATGACGTTCGTGTTGTTTTAGA-3’  Reverse : 5’-CTGAGGGTCCACCAAACGTA-3’ | 6FAM-GTCTCTAAATGGACCCCAAAATCA | | 110 |
| Delta variant | S-Gene  (Mutation P681R) | Sense : 5’-TGACATACCCATTGGTGCAG-3’  Reverse : 5’-GGCAATGATGGATTGACTAGC-3’ | 6FAM-ATTCTCGTCGGCGGGCA | | 97 |
| Beta variant | nsp2 | Sense : 5’-TGAATTGCAGACACCTTTTGA-3’  Reverse : 5’-CAACCCTTGGTTGAATAGTCTTG-3’ | 6FAM-TGACATCTTCAATGGGGAATGT | | 119 |
| Gamma variant | N-Gene | Sense : 5’-GTCAAGCCTCTTCTCGTTCCT-3’  Reverse : 5’-AAAGCAAGAGCAGCATCACC-3’ | 6FAM-GCAGCTCTAAACGAACTTCTCCTG | | 124 |
| Wuhan | RdRp-Gene | Sense : 5’-GCATTCTGCATTGTGCAAAC-3’  Reverse : 5’-ACAAATGGAACACCATCAACAA-3’ | 6FAM-CCCACCTACAAGTTTTGGACC | | 106 |
| Wuhan | S-Gene  (614D) | Sense : 5’-AACCAGGTTGCTGTTCTTTATCA-3’  Reverse : 5’-CACGCCAAGTAGGAGTAAGTTGA-3’ | 6FAM-ATCAGGATGTTAACTGCACAGAAGT | | 88 |
| Marseille 4 variant  (Lineage B.1.160) | ORF1 | Sense : 5’-GAGGTTTAGAAGAGCTTTTGGTGA-3’  Reverse : 5’-CCAGGTAAGAATGAGTAAACTGGTG-3’ | 6FAM-GCAACATTTCAAGAAATTCAACTC | | 124 |
| Marseille 1 variant  (Lineage B.1.416) | ORF1 | Sense : 5’-TCAAGCCTCTTCTCGTTCCTT-3’  Reverse : 5’-CAAAGCAAGAGCAGCATCAC-3’ | 6FAM-GCAACATTTCAAGAAATTCAACTC | | 124 |

6-FAM : 6-carboxyfluorescein, bp : base pair

Supplementary Table 2 : Samples ID, GISAID accession ID and Lineage

| Sample ID | GISAID Accession ID | Lineage | qPCR genotyping |
| --- | --- | --- | --- |
| 34 | EPI_ISL_8886131 | B.1 |  |
| 56 | EPI_ISL_8886132 | A |  |
| 62 | EPI_ISL_8886133 | B.1.356 |  |
| 7919 | EPI_ISL_8886134 | B.1.1 |  |
| 7921 | EPI_ISL_8886135 | B.1.1 |  |
| 11046 | EPI_ISL_8886136 | B.1.617.2 |  |
| 2056-AC1 | EPI_ISL_8886137 | B.1.1 |  |
| 3841-NNO1 | EPI_ISL_8886138 | B.1.1 |  |
| 3842-AF1 | EPI_ISL_8886139 | B.1.1 |  |
| 5906-NMD1 | EPI_ISL_8886140 | B.1.1 |  |
| 5901-YPB1 | EPI_ISL_8886141 | B.1.1 |  |
| 5789-ENM1 | EPI_ISL_8886142 | B.1.1 |  |
| 6739-IKJ1 | EPI_ISL_8886143 | B.1.1 |  |
| 5891-BA1 | EPI_ISL_8886144 | B.1.1 |  |
| 7562-KS1 | EPI_ISL_8886145 | B.1.1.121 |  |
| 1300-2A | EPI_ISL_8886146 | B.1.1.409 | Negative VOCs |
| 1301-3A | EPI_ISL_8886147 | B.1.1 | Negative VOCs |
| 1306-5A | EPI_ISL_8886148 | B.1.1 | Negative VOCs |
| 1307-6A | EPI_ISL_8886149 | B.1.1 | Negative VOCs |
| 1318-10A | EPI_ISL_8886150 | B.1.1 | Negative VOCs |
| 1340-11A | EPI_ISL_8886151 | B.1.1 | Negative VOCs |
| 1344-12A | EPI_ISL_8886152 | B.1.1 | Negative VOCs |
| 1362-14A | EPI_ISL_8886153 | B.1.1 | Negative VOCs |
| 1386-15A | EPI_ISL_8886154 | B.1.1 | Negative VOCs |
| 1412-16A | EPI_ISL_8886155 | B.1.1 | Negative VOCs |
| 1455-17A | EPI_ISL_8886156 | B.1.1 | Negative VOCs |
| 1459-18A | EPI_ISL_8886157 | B.1.1 | Negative VOCs |
| 1460-19A | EPI_ISL_8886158 | B.1.1 | Negative VOCs |
| 1462-20A | EPI_ISL_8886159 | B.1.1 | Negative VOCs |
| 1465-21A | EPI_ISL_8886160 | B.1.1 | Negative VOCs |
| 1475-22A | EPI_ISL_8886161 | B.1.1 | Negative VOCs |
| 1479-23A | EPI_ISL_8886162 | B.1.1 | Negative VOCs |
| 1488-24A | EPI_ISL_8886163 | B.1.1 | Negative VOCs |
| 1491-25A | EPI_ISL_8886164 | B.1.1 | Negative VOCs |
| 1539-26A | EPI_ISL_8886165 | B.1.1.409 | Negative VOCs |
| 1701-27A | EPI_ISL_8886166 | B.1.1 | Negative VOCs |
| 1766-30A | EPI_ISL_8886167 | B.1.1 | Negative VOCs |
| 1782-31A | EPI_ISL_8886168 | B.1.1 | Negative VOCs |
| 1832-32A | EPI_ISL_8886169 | B.1.1 | Negative VOCs |
| 1833-33A | EPI_ISL_8886170 | B.1.1 | Negative VOCs |
| 1834-34A | EPI_ISL_8886171 | B.1.1 | Negative VOCs |
| 1836-35A | EPI_ISL_8886172 | B.1.1 | Negative VOCs |
| 1879-36A | EPI_ISL_8886173 | B.1.1 | Negative VOCs |
| 1920-38A | EPI_ISL_8886174 | B.1.1 | Negative VOCs |
| 2018-41A | EPI_ISL_8886175 | B.1.1 | Negative VOCs |
| 64L | EPI_ISL_8886176 | B.1.128 |  |
| 88L | EPI_ISL_8886177 | B.1.128 |  |
| 101L | EPI_ISL_8886178 | B.1.128 |  |
| 149L | EPI_ISL_8886179 | B.1.1 |  |
| 196L | EPI_ISL_8886180 | B.1.1 |  |
| 211L | EPI_ISL_8886181 | A |  |
| 266L | EPI_ISL_8886182 | B.1.1.275 |  |
| 557L | EPI_ISL_8886183 | B.1.1 |  |
| 560L | EPI_ISL_8886184 | B.1.128 |  |
| 567L | EPI_ISL_8886185 | B.1.1 |  |
| 617L | EPI_ISL_8886186 | B.1.1 |  |
| 627L | EPI_ISL_8886187 | B.1.1 |  |
| 631L | EPI_ISL_8886188 | B.1.1 |  |
| 636L | EPI_ISL_8886190 | B.1.1 |  |
| 1740L | EPI_ISL_8886191 | B.1.1 |  |
| 3675L | EPI_ISL_8886192 | B.1.1 |  |
| 3803L | EPI_ISL_8886193 | B.1.1 |  |
| 3815L | EPI_ISL_8886194 | B.1.1 |  |
| 4035L | EPI_ISL_8886195 | B.1.1 |  |
| 4045L | EPI_ISL_8886196 | B.1.1.275 |  |
| 12981 | EPI_ISL_8886197 | B.1 |  |
| 12982 | EPI_ISL_8886198 | B.1 |  |
| 13627 | EPI_ISL_8886199 | B.1 |  |
| 16601 | EPI_ISL_8886200 | B.1 |  |
| 12245 | EPI_ISL_8886201 | L.3 |  |
| 12285 | EPI_ISL_8886202 | L.3 |  |
| 12388 | EPI_ISL_8886203 | L.3 |  |
| 12833 | EPI_ISL_8886204 | L.3 |  |
| 12451 | EPI_ISL_8886205 | B.1.214.3 |  |
| 13313 | EPI_ISL_8886206 | B.1.214.3 |  |
| 13593 | EPI_ISL_8886207 | B.1.214.3 |  |
| 16143 | EPI_ISL_8886208 | B.1.214.2 |  |
| 12928 | EPI_ISL_8886209 | B.1.1.7 |  |
| 12929 | EPI_ISL_8886210 | B.1.1.7 |  |
| 13123 | EPI_ISL_8886211 | B.1.1.7 |  |
| 13134 | EPI_ISL_8886212 | B.1.1.7 |  |
| 13171 | EPI_ISL_8886213 | B.1.1.7 |  |
| 13307 | EPI_ISL_8886214 | B.1.1.7 |  |
| 13350 | EPI_ISL_8886215 | B.1.1.7 |  |
| 13351 | EPI_ISL_8886216 | B.1.1.7 |  |
| 13406 | EPI_ISL_8886217 | B.1.1.7 |  |
| 13416 | EPI_ISL_8886218 | B.1.1.7 |  |
| 13417 | EPI_ISL_8886219 | B.1.1.7 |  |
| 13441 | EPI_ISL_8886220 | B.1.1.7 |  |
| 13452 | EPI_ISL_8886221 | B.1.1.7 |  |
| 13464 | EPI_ISL_8886222 | B.1.1.7 |  |
| 13506 | EPI_ISL_8886223 | B.1.1.7 |  |
| 13623 | EPI_ISL_8886225 | B.1.1.7 |  |
| 13824 | EPI_ISL_8886226 | B.1.1.7 |  |
| 14428 | EPI_ISL_8886227 | B.1.1.7 |  |
| 14945 | EPI_ISL_8886228 | B.1.1.7 |  |
| 14946 | EPI_ISL_8886229 | B.1.1.7 |  |
| 15097 | EPI_ISL_8886230 | B.1.1.7 |  |
| 15139 | EPI_ISL_8886231 | B.1.1.7 |  |
| 15141 | EPI_ISL_8886232 | B.1.1.7 |  |
| 15380 | EPI_ISL_8886233 | B.1.1.7 |  |
| 15489 | EPI_ISL_8886234 | B.1.1.7 |  |
| 15439 | EPI_ISL_8886235 | B.1.1.7 |  |
| 15502 | EPI_ISL_8886236 | B.1.1.7 |  |
| 15679 | EPI_ISL_8886237 | B.1.1.7 |  |
| 16138 | EPI_ISL_8886238 | B.1.1.7 |  |
| 16686 | EPI_ISL_8886239 | B.1.1.7 |  |
| 16620 | EPI_ISL_8886240 | B.1.1.7 |  |
| 16622 | EPI_ISL_8886241 | B.1.1.7 |  |
| 18041 | EPI_ISL_8886242 | B.1.1.7 |  |
| 18046 | EPI_ISL_8886243 | B.1.1.7 |  |
| 21363 | EPI_ISL_8886244 | B.1.1.7 |  |
| 14017-1B | EPI_ISL_8886245 | B.1.1.7 | VOC Alpha |
| 14403-2B | EPI_ISL_8886246 | B.1.1.7 | VOC Alpha |
| 14426-3B | EPI_ISL_8886247 | B.1.1.7 | VOC Alpha |
| 14433-4B | EPI_ISL_8886248 | B.1.1.7 | VOC Alpha |
| 14434-5B | EPI_ISL_8886249 | B.1.1.7 | VOC Alpha |
| 14453-6B | EPI_ISL_8886250 | B.1.1.7 | VOC Alpha |
| 14537-7B | EPI_ISL_8886251 | B.1.214.2 |  |
| 14579-8B | EPI_ISL_8886252 | B.1.1.7 | VOC Alpha |
| 14580-9B | EPI_ISL_8886253 | B.1.1.7 | VOC Alpha |
| 14606-10B | EPI_ISL_8886254 | B.1.1.7 | VOC Alpha |
| 14617-11B | EPI_ISL_8886255 | B.1.1.7 | VOC Alpha |
| 14637-12B | EPI_ISL_8886256 | B.1.1.7 | VOC Alpha |
| 14648-13B | EPI_ISL_8886257 | B.1.1.7 | VOC Alpha |
| 14672-14B | EPI_ISL_8886258 | B.1.1.7 | VOC Alpha |
| 14687-15B | EPI_ISL_8886259 | B.1.1.7 | VOC Alpha |
| 14688-16B | EPI_ISL_8886260 | B.1.1.7 | VOC Alpha |
| 14696-17B | EPI_ISL_8886261 | B.1.1.7 | VOC Alpha |
| 14697-18B | EPI_ISL_8886262 | B.1.214.2 |  |
| 14698-19B | EPI_ISL_8886263 | B.1.1.7 | VOC Alpha |
| 14750-20B | EPI_ISL_8886264 | B.1.1.7 | VOC Alpha |
| 14752-21B | EPI_ISL_8886265 | B.1.1.7 | VOC Alpha |
| 14767-22B | EPI_ISL_8886266 | B.1.1.7 | VOC Alpha |
| 14780-23B | EPI_ISL_8886267 | B.1.1.7 | VOC Alpha |
| 14782-24B | EPI_ISL_8886268 | B.1.1.7 | VOC Alpha |
| 14794-25B | EPI_ISL_8886269 | B.1.1.7 | VOC Alpha |
| 14795-26B | EPI_ISL_8886270 | B.1.1.7 | VOC Alpha |
| 14796-27B | EPI_ISL_8886271 | B.1.1.7 | VOC Alpha |
| 14808-28B | EPI_ISL_8886272 | B.1.214.3 |  |
| 14817-29B | EPI_ISL_8886273 | B.1.1.7 | VOC Alpha |
| 14818-30B | EPI_ISL_8886274 | B.1.1.7 | VOC Alpha |
| 14826-32B | EPI_ISL_8886275 | B.1.214.2 |  |
| 14829-33B | EPI_ISL_8886276 | B.1.1.7 | VOC Alpha |
| 14837-34B | EPI_ISL_8886277 | B.1.1.7 | VOC Alpha |
| 14868-35B | EPI_ISL_8886278 | B.1.1.7 | VOC Alpha |
| 14883-36B | EPI_ISL_8886279 | B.1.1.7 | VOC Alpha |
| 14887-37B | EPI_ISL_8886280 | B.1.1.7 | VOC Alpha |
| 4716 | EPI_ISL_8886281 | B.1.1.7 |  |
| 4718 | EPI_ISL_8886282 | B.1.1.318 |  |
| 4721 | EPI_ISL_8886283 | B.1.1.318 |  |
| 4801 | EPI_ISL_8886284 | B.1.1.318 |  |
| 4804 | EPI_ISL_8886285 | B.1.1.318 |  |
| 4859 | EPI_ISL_8886286 | B.1.1.7 |  |
| 4869 | EPI_ISL_8886287 | B.1.1.318 |  |
| 4877 | EPI_ISL_8886288 | B.1.1.318 |  |
| 4897 | EPI_ISL_8886289 | B.1.1.318 |  |
| 4898 | EPI_ISL_8886290 | B.1.1.318 |  |
| 4906 | EPI_ISL_8886291 | B.1.1.7 |  |
| 4907 | EPI_ISL_8886292 | B.1.1.318 |  |
| 4908 | EPI_ISL_8886293 | B.1 |  |
| 4910 | EPI_ISL_8886294 | B.1.1.318 |  |
| 4916 | EPI_ISL_8886295 | B.1.1.7 |  |
| 4927 | EPI_ISL_8886297 | B.1.1.318 |  |
| 4942 | EPI_ISL_8886298 | B.1.1.318 |  |
| 4963 | EPI_ISL_8886299 | B.1.1.318 |  |
| 4968L | EPI_ISL_8886300 | B.1.1.318 |  |
| 4978 | EPI_ISL_8886301 | B.1.1.318 |  |
| 4986 | EPI_ISL_8886302 | B.1.1.318 |  |
| 5006 | EPI_ISL_8886303 | B.1.1.318 |  |
| 5013 | EPI_ISL_8886304 | B.1.1.318 |  |
| 5023 | EPI_ISL_8886305 | B.1.1.318 |  |
| 5031 | EPI_ISL_8886306 | B.1.1.318 |  |
| 5033 | EPI_ISL_8886307 | B.1.525 |  |
| 5036 | EPI_ISL_8886308 | B.1.1.318 |  |
| 5039 | EPI_ISL_8886309 | B.1.1.318 |  |
| 5067 | EPI_ISL_8886310 | B.1.1.318 |  |
| 5074 | EPI_ISL_8886311 | B.1.1.7 |  |
| 5079 | EPI_ISL_8886312 | B.1.1.318 |  |
| 5090 | EPI_ISL_8886313 | B.1.1.318 |  |
| 5129 | EPI_ISL_8886314 | B.1.1.7 |  |
| 5131 | EPI_ISL_8886315 | B.1.1.318 |  |
| 5156 | EPI_ISL_8886316 | B.1.1.7 |  |
| 5161 | EPI_ISL_8886317 | B.1.1.318 |  |
| 5228 | EPI_ISL_8886318 | B.1.1.318 |  |
| 5236 | EPI_ISL_8886319 | B.1.1.318 |  |
| 5254 | EPI_ISL_8886320 | B.1.1.318 |  |
| 5262 | EPI_ISL_8886321 | B.1.1.318 |  |
| 5299 | EPI_ISL_8886322 | B.1.1.318 |  |
| 5302 | EPI_ISL_8886323 | B.1.1.318 |  |
| 5317 | EPI_ISL_8886324 | B.1.1.318 |  |
| 5329 | EPI_ISL_8886325 | B.1.1.318 |  |
| 5382 | EPI_ISL_8886326 | B.1.1.318 |  |
| 5407 | EPI_ISL_8886327 | B.1.1.318 |  |
| 5414 | EPI_ISL_8886328 | B.1.1.318 |  |
| 5469 | EPI_ISL_8886329 | B.1.1.318 |  |
| 5472 | EPI_ISL_8886330 | B.1.1.318 |  |
| 5475 | EPI_ISL_8886331 | B.1.1.318 |  |
| 5547 | EPI_ISL_8886332 | B.1.1.318 |  |
| 5573 | EPI_ISL_8886333 | B.1.1.318 |  |
| 5590 | EPI_ISL_8886334 | B.1.1.318 |  |
| 5619 | EPI_ISL_8886335 | B.1.1.318 |  |
| 5625 | EPI_ISL_8886336 | B.1.1.318 |  |
| 5652 | EPI_ISL_8886337 | B.1.620 |  |
| 22849-1C | EPI_ISL_8886338 | B.1.617.2 | VOC Delta |
| 24051-2C | EPI_ISL_8886339 | B.1.617.2 | VOC Delta |
| 24130-3C | EPI_ISL_8886340 | B.1.617.2 | VOC Delta |
| 24138-4C | EPI_ISL_8886341 | B.1.617.2 | VOC Delta |
| 24141-5C | EPI_ISL_8886342 | B.1.617.2 | VOC Delta |
| 24182-6C | EPI_ISL_8886343 | B.1.617.2 | VOC Delta |
| 24211-8C | EPI_ISL_8886344 | B.1.617.2 | VOC Delta |
| 24246-9C | EPI_ISL_8886345 | B.1.617.2 | VOC Delta |
| 24250-10C | EPI_ISL_8886346 | B.1.617.2 | VOC Delta |
| 24268-11C | EPI_ISL_8886347 | B.1.617.2 | VOC Delta |
| 24295-12C | EPI_ISL_8886348 | B.1.617.2 | VOC Delta |
| 24307-13C | EPI_ISL_8886349 | B.1.617.2 | VOC Delta |
| 24385-14C | EPI_ISL_8886350 | B.1.617.2 | VOC Delta |
| 24785-15C | EPI_ISL_8886351 | B.1.617.2 | VOC Delta |
| 24823-17C | EPI_ISL_8886352 | B.1.617.2 | VOC Delta |
| 24830-18C | EPI_ISL_8886353 | B.1.617.2 | VOC Delta |
| 24870-19C | EPI_ISL_8886354 | B.1.617.2 | VOC Delta |
| 24885-20C | EPI_ISL_8886355 | B.1.617.2 | VOC Delta |
| 24920-21C | EPI_ISL_8886356 | B.1.617.2 | VOC Delta |
| 24922-22C | EPI_ISL_8886357 | B.1.617.2 | VOC Delta |
| 24945-23C | EPI_ISL_8886358 | B.1.617.2 | VOC Delta |
| 24961-24C | EPI_ISL_8886359 | B.1.617.2 | VOC Delta |
| 24966-25C | EPI_ISL_8886360 | B.1.617.2 | VOC Delta |
| 25012-27C | EPI_ISL_8886361 | B.1.617.2 | VOC Delta |
| 25014-28C | EPI_ISL_8886362 | B.1.617.2 | VOC Delta |
| 25020-29C | EPI_ISL_8886363 | B.1.617.2 | VOC Delta |
| 25021-30C | EPI_ISL_8886364 | B.1.617.2 | VOC Delta |
| 25025-31C | EPI_ISL_8886365 | B.1.617.2 | VOC Delta |
| 25026-32C | EPI_ISL_8886366 | B.1.617.2 | VOC Delta |
| 25027-33C | EPI_ISL_8886367 | B.1.617.2 | VOC Delta |
| 25068-34C | EPI_ISL_8886368 | B.1.617.2 | VOC Delta |
| 25069-35C | EPI_ISL_8886369 | B.1.617.2 | VOC Delta |
| 24830-RY4 | EPI_ISL_8886370 | B.1.617.2 |  |
| 22044 | EPI_ISL_8886371 | B.1.1.318 |  |
| 5729 | EPI_ISL_8886372 | B.1.620 |  |
| 5772 | EPI_ISL_8886373 | B.1.1.318 |  |
| 5870 | EPI_ISL_8886374 | B.1.1.318 |  |
| 6242 | EPI_ISL_8886375 | B.1.1.318 |  |
| 6287 | EPI_ISL_8886376 | B.1.1.318 |  |
| 6709 | EPI_ISL_8886377 | B.1.1.318 |  |
| 6776 | EPI_ISL_8886378 | B.1.1.318 |  |
| 6788 | EPI_ISL_8886379 | B.1.1.318 |  |
| 6829 | EPI_ISL_8886380 | B.1.1.318 |  |
| 6902L | EPI_ISL_8886381 | B.1.1.318 |  |
| 7003 | EPI_ISL_8886382 | B.1.617.2 |  |
| 7004 | EPI_ISL_8886383 | B.1.617.2 |  |
| 7084 | EPI_ISL_8886384 | B.1.617.2 |  |
| 7085 | EPI_ISL_8886385 | B.1.617.2 |  |
| 7086 | EPI_ISL_8886386 | B.1.617.2 |  |
| 7166 | EPI_ISL_8886387 | B.1.617.2 |  |
| 7177 | EPI_ISL_8886388 | B.1.617.2 |  |
| 7191 | EPI_ISL_8886389 | B.1.617.2 |  |
| 7192 | EPI_ISL_8886390 | B.1.617.2 |  |
| 7203 | EPI_ISL_8886391 | B.1.617.2 |  |
| 7211 | EPI_ISL_8886392 | B.1.617.2 |  |
| 7222 | EPI_ISL_8886393 | B.1.617.2 |  |
| 7257 | EPI_ISL_8886394 | B.1.617.2 |  |
| 7260 | EPI_ISL_8886395 | B.1.617.2 |  |
| 7268L | EPI_ISL_8886396 | B.1.617.2 |  |
| 7279L | EPI_ISL_8886397 | B.1.617.2 |  |
| 7282 | EPI_ISL_8886398 | B.1.617.2 |  |
| 7283L | EPI_ISL_8886399 | B.1.617.2 |  |
| 7286L | EPI_ISL_8886400 | B.1.617.2 |  |
| 7292 | EPI_ISL_8886401 | B.1.617.2 |  |
| 7293 | EPI_ISL_8886402 | B.1.617.2 |  |
| 7298L | EPI_ISL_8886404 | B.1.617.2 |  |
| 7324L | EPI_ISL_8886405 | B.1.617.2 |  |
| 7341L | EPI_ISL_8886406 | B.1.617.2 |  |
| 7343 | EPI_ISL_8886407 | B.1.617.2 |  |
| 7348L | EPI_ISL_8886408 | B.1.617.2 |  |
